# Paying it forward: Crowdsourcing the harmonisation and linking of taxon names and biodiversity identifiers

**DOI:** 10.3897/BDJ.11.e114076

**Published:** 2023-11-24

**Authors:** Brandon Kwee Boon Seah

**Affiliations:** 1 Thünen Institute for Biodiversity, Braunschweig, Germany Thünen Institute for Biodiversity Braunschweig Germany

**Keywords:** data curation, biodiversity informatics, data integration

## Abstract

Linking records for the same taxa between different databases is an essential step when working with biodiversity data. However, name-matching alone is error-prone, because of issues such as homonyms (unrelated taxa with the same name) and synonyms (same taxon under different names). Therefore, most projects will require some curation to ensure that taxon identifiers are correctly linked. Unfortunately, formal guidance on such curation is uncommon and these steps are often ad hoc and poorly documented, which hinders transparency and reproducibility, yet the task requires specialist knowledge and cannot be easily automated without careful validation. Here, we present a case study on linking identifiers between the GBIF and NCBI taxonomies for a species checklist. This represents a common scenario: finding published sequence data (from NCBI) for species chosen by occurrence or geographical distribution (from GBIF). Wikidata, a publicly editable knowledge base of structured data, can serve as an additional information source for identifier linking. We suggest a software toolkit for taxon name-matching and data-cleaning, describe common issues encountered during curation and propose concrete steps to address them. For example, about 2.8% of the taxa in our dataset had wrong identifiers linked on Wikidata because of errors in name-matching caused by homonyms. By correcting such errors during data-cleaning, either directly (through editing Wikidata) or indirectly (by reporting errors in GBIF or NCBI), we crowdsource the curation and contribute to community resources, thereby improving the quality of downstream analyses.

## Introduction

Biodiversity science has seen a proliferation of databases and checklists (Feng et al. 2022). While taxonomic experts are best-placed to curate data for their respective taxa of expertise, there are drawbacks to group-specific, specialised databases: they may not be maintained in the long term, may not be interoperable with other databases and may duplicate efforts when different projects have overlapping coverage or aims (Schellenberger Costa et al. 2023). Similar observations have been made about the software developed for working with them (Grenié et al. 2021). As a result, users face the challenge of integrating different databases by linking or harmonising taxon names and database-specific identifiers, before they can take advantage of the domain-specific information contained in them.

End-users can match taxa either by their names or taxon identifiers. This task is a subset of data reconciliation or data matching (Christen 2012), a dynamic field with evolving standards (Delpeuch et al. 2023). Some databases, particularly those that themselves aggregate multiple sources (“data aggregators”), may incorporate cross-references to other databases, but end-users are ultimately responsible for curating the data they wish to use and often have to rely on name-matching. The Linnaean system has been in use for almost three centuries, which attests to its utility and robustness, but names are human artefacts and, hence, inherently prone to variants (e.g. in orthography) and errors (Patterson et al. 2016). Additionally, a given name may also embody different taxon concepts (cf. International Commission on Zoological Nomenclature (1999) Article 61.3; Turland et al. (2018) Glossary).

How can we avoid duplicated effort in data curation? Ideally, users of taxonomic data would share in building and improving community resources, as they are often also the subject-matter experts. Building yet another database is clearly not the answer. Nonetheless, large aggregator projects, such as WoRMS and ITIS, tend to be centrally organised and may not have a formal avenue for user contributions. Wikidata (https://www.wikidata.org/) (Vrandečić and Krötzsch 2014) presents an alternative model for how data curation can be crowdsourced. Like Wikipedia, its better-known cousin, Wikidata is freely accessible and editable by online users and is actually the backend for many automatically generated information boxes displayed in Wikipedia articles, for example, for biological taxa (https://en.wikipedia.org/wiki/Template:Taxonbar). The Wikidata project aims to build a general knowledge graph, comprising items (entities or objects of any kind, including abstract concepts) linked by statements about their relationships. Each statement comprises a subject and an object (items) linked by a predicate (a property). For example, the item “*Coffeaarabica*” (Q47685) is linked to the item “coffee bean” (Q153697) by the property “this taxon is source of” (P1672). Biological taxa are modelled as instances of (P31) taxon (Q16521) and typically have properties like taxon name (P225), authors (P405) and rank (P105). Taxon identifiers in other databases can be represented through statements where the object is not a Wikidata item, but a text string, for example “*Coffeaarabica*” (Q47685) has a property “GBIF taxon ID” (P846) with the value “2895345”.

Page (2022) has argued that it is ultimately more productive and sustainable to contribute to an existing project already supported by an active community, such as Wikidata, than to start a new domain-specific project, where such a user base would have to be built up from scratch. Wikidata is already used in the life sciences for purposes such as crowdsourcing biological ontologies and data-mining for drug discovery and disease diagnosis (Waagmeester et al. 2020) or natural products chemistry (Rutz et al. 2022). In biodiversity informatics, it has been proposed as a platform for a “bibliography of life” — a comprehensive linked database of the taxonomic literature (Page 2022) and to disambiguate personal names in collection records (Groom et al. 2022).

Graphs of database identifiers have been used instead of name-matching to link over a hundred thousand entries in Wikidata with the Global Biotic Interactions Database (GloBI) (Thessen et al. 2018). These large numbers are impressive, but rely on the identifiers being up-to-date and correctly assigned. As a crowdsourced platform, the accuracy of Wikidata depends on smaller, individual contributions. If one is not solely interested in global patterns, but also specific cases, then careful curation is necessary. This more modest, but ultimately essential “bricklaying” by individual users is the topic of this case study.

Here, we describe how we match taxon names and identifiers between the Global Biodiversity Information Facility (GBIF) Backbone Taxonomy (GBIF Secretariat 2022) and the NCBI Taxonomy (Schoch et al. 2020), integrating Wikidata into the workflow both as a source of linked identifiers to speed up data-matching and as a community resource that we contribute to during data curation. GBIF aggregates biodiversity distribution and occurrence data, whereas the main international repositories for molecular sequence data, the International Nucleotide Sequence Database Collaboration (INSDC), of which NCBI is a member, use the NCBI Taxonomy. This represents a common usage scenario of finding biological sequences that belong to a set of taxa. The dataset used is a checklist of vascular plants from Germany (Bundesamt für Naturschutz 2021). As this is a region well-studied by botanists, we expect that virtually all species have been described and that most are well documented with published occurrence and sequence data.

Our aims are to identify issues commonly encountered during data-matching, in particular the actual impact of homonymy and synonymy on name-matching and to make concrete suggestions for how to troubleshoot and improve community resources as part of the data cleaning process, as a form of crowdsourcing.

## Description and implementation

The three databases model the relationships between taxa and taxon names differently. Taxon names are formally governed by rules of nomenclature, which decide whether they are validly published. However the circumscription and classification of the taxon concept referred to by a given name can be a matter of legitimate scientific (taxonomic) disagreement. The GBIF Backbone and NCBI Taxonomy explicitly designate a preferred taxonomy. When more than one name is thought to represent the same taxon, GBIF and NCBI explicitly choose one name as accepted and mark the others as synonyms. In GBIF, each name has a distinct taxon identifier (taxonID) and a taxonomic status (e.g. “accepted”, “synonym”), whereas, in NCBI, records for synonyms are merged into the taxonID of the accepted name and the former taxonIDs of the synonyms are deprecated. In Wikidata, each taxon name is a distinct item like in GBIF, but there is no preferred taxonomy. Items representing synonyms can be linked by the property “taxon synonym” (P1420), ideally citing a reference where this relationship is asserted.

### Workflow to link identifiers and flag cases for curation

The dataset (https://doi.org/10.15468/0fxsox) comprises 7209 taxon names of vascular plants from Germany (5876 at species rank) and their associated GBIF taxonIDs which we wished to link to equivalent NCBI Taxonomy taxonIDs. The file was downloaded from GBIF as a “species list”, which lists taxa in a tab-separated text file, containing the taxon name as supplied by the data provider, the taxonID for that name, the “accepted” taxon in the GBIF Backbone Taxonomy to which it was matched when the dataset was imported, its taxonomic status and taxon rank and the names and taxonIDs of the higher taxa to which it belongs (kingdom, phylum etc.).

For reproducibility, we used flatfiles of the latest available versions of the GBIF Backbone Taxonomy (26 Nov 2021) and the NCBI Taxonomy (01 Dec 2022) instead of live online queries, so that the analysis could be pinned to a specific version as these databases are continuously updated. For Wikidata, we directly queried the online API instead of downloading a versioned flatfile, because database dump files are large (23 Jun 2023 version over 136 GB) and contain data on all entities, not just biological taxonomy; queries can also be submitted via the web interface, either in the SPARQL query language or with the interactive query builder and the results exported as a table.

GBIF taxonIDs in the dataset were matched against the GBIF Backbone Taxonomy to filter out records that have been marked as “doubtful” or problematic and to find currently accepted names and taxonIDs within the GBIF Backbone Taxonomy, as the latter may have been updated after the dataset was originally imported. This resulted in a table of taxon names (with authors) and taxonIDs of interest. Only taxa of species rank (5721 names) were retained to simplify the search, as the higher taxa can be derived from the list of species. From the NCBI Taxonomy, scientific names (including authors where available) and taxonIDs at species rank classified to Viridiplantae (NCBI:txid33090) were retrieved, to reduce the number of names to be searched and to avoid hemihomonyms.

**Figure 1.**
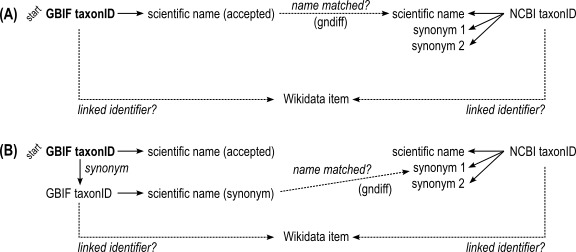
Simplified diagram of identifier linking through name matching. **A** Match accepted taxon names in GBIF against names in NCBI Taxonomy using gndiff, then check if the respective identifiers are also linked in Wikidata; **B** If an accepted name had no matches, retrieve synonyms for a second round of name matching.

The GBIF taxon names were matched against the Viridiplantae taxon names from NCBI with Gndiff v.0.2.0 (https://github.com/gnames/gndiff) (Fig. 1 panel A), which matches taxon names while accounting for common orthographic variants, errors and other issues specific to taxon names. Gndiff uses the same algorithms as Gnverifier (https://doi.org/10.5281/zenodo.5111542) and Gnparser (Mozzherin et al. 2017), but can be used offline and without an external database. Gndiff reports three types of matches: “Exact”, “PartialExact”, “Fuzzy”. We excluded “PartialExact” matches because they encompass cases where only the genus name matches. “Fuzzy” matches include potential misspellings and so were retained. Gndiff parses the author field, if present, but does not take them into account, so we further classified “Exact” matches into three types, based on the author names: “exact” – author names or citations identical, “noauthor” – author names absent from one or both entries (typically from the NCBI record), “author_mismatch” – author names do not match exactly, which includes differences in abbreviation or orthography. The result was a table of GBIF taxonIDs linked to NCBI taxonIDs by name matching.

For GBIF names without matches in NCBI Taxonomy, synonyms according to the GBIF Backbone were retrieved and then used for a second round of name-matching (Fig. 1 panel B). This was to account for cases where the same taxon has different accepted names in the two databases.

We queried Wikidata via its SPARQL API (https://query.wikidata.org/) for taxon items with the GBIF taxonIDs from our dataset (property P846). If they were linked to an NCBI taxonID (property P685), the linked NCBI taxonID was added to our table. If a taxon name were not linked to a Wikidata item via its GBIF taxonID, but the earlier name matching had found an NCBI taxonID, then the NCBI taxonID was used to query Wikidata to find linked Wikidata items and their associated GBIF taxonIDs, if available.

The identifier links on Wikidata were then used to categorise the pairs of matched names for further action (Table 1). The aim was to filter out names with no matches (Table 1, curation action “a, nothing more to be done”) or unambiguous matches (Table 1, curation action “b, automatically accepted”) from cases needing additional curation.

We identified straightforward cases of missing or outdated information in Wikidata, which can be updated through batch edits (Table 1, curation actions d and e). The criteria were that GBIF and NCBI names had an exact match (including authorship) and the name was accepted in the GBIF Backbone, but Wikidata either did not have one of the taxonIDs or had a different taxonID from the currently accepted one. Commands for executing batch edits with the QuickStatements tool (https://quickstatements.toolforge.org/) were generated.

To understand the underlying causes for these erroneous links, we further investigated the cases where name-matching and Wikidata disagree on the GBIF taxonID (Table 1, curation action e). The current taxonomic status of the GBIF taxonIDs found in Wikidata was looked up in the GBIF Backbone Taxonomy. Of the 245 taxonIDs, two cases represented mismatched ranks (one genus and one subspecies) and another 83 (1.5% of 5721 total) had been removed by GBIF curators and were no longer listed in the GBIF Backbone, but these updates were not yet propagated to Wikidata. Almost all the remaining 162 (2.8%) appear to be names wrongly matched when identifiers were added to Wikidata because of homonymy, because the taxon authors differ.

The remaining cases were then tabulated for manual curation. This requires some knowledge of taxonomy and nomenclature rules to be able to evaluate whether two names are equivalent or not, as well as cross-checking against additional databases.

### Guide to manual curation and improving community resources

Here, we describe what issues can be found during manual curation and what concrete action users can take to improve the database resources. In brief: Wikidata can be edited directly to fix errors or add missing information, preferably after creating a user account; issues with the GBIF Backbone Taxonomy can be reported via the website feedback dialogue, by email or via Github; issues with the NCBI Taxonomy should be reported by email.

#### Errors due to name matching

Error modes in name matching have been extensively discussed (Patterson et al. 2016, Remsen 2016). In the curation process, homonyms can be quickly recognised by mismatches in authorships; those links can be rejected unless they are simply orthographic differences, such as the removal of diacritics (e.g. “Hultén” vs. “Hulten”) or different abbreviation conventions (“Hook.f.” vs. “Hook.fil.”). Typographical errors are to some extent ameliorated by the fuzzy matching in Gndiff.

**Example**: Name-matching errors may also appear in the source databases. The original dataset listed *Ammophila* Kirby, 1798 (GBIF taxonID 1346141), a genus of wasps, instead of the grass genus *Ammophila* Host (GBIF 2703794). Both names are valid under their respective, independent nomenclatural codes, i.e. they are hemihomonyms. Here, the error appears to have occurred during import of the data from the original provider into GBIF.

**Action**: Accept or reject the linked identifiers after verification.

#### Errors or information gaps in databases

If the results of name matching disagree with database identifiers, it is possible that one or more of the source databases have incomplete or erroneous information.

*1. GBIF taxonID has been deprecated or merged*.

The GBIF Backbone Taxonomy is continually revised and records may be deleted if they are, for example, doubtful names, orthographic errors or duplicates. However, the deprecated GBIF taxonIDs may still be linked in Wikidata. In some cases, the accepted taxon in GBIF may also be in error (see point 6 below).

**Example**: The Wikidata record for *Helianthusannuus* (Q171497) was linked to the GBIF taxonID 3119195, which was deleted on 01 Feb 2018. The currently accepted GBIF record for this species is 9206251.

**Action**: When unambiguous, edit the Wikidata entry to add the currently accepted GBIF taxon, after checking that it is not a homonym. Record the access date in the reference with the property “retrieved” (P813), which will help future editors troubleshoot if the GBIF record changes again. The outdated GBIF identifier value can be explicitly marked with a "deprecated" rank, with a qualifier stating that the reason (P2241) is that the identifier was deprecated in the source database (Q67125514). See Shafee et al. (2023) for guidance on editing Wikidata.

*2. NCBI taxonID has been deprecated or merged*.

Unlike GBIF, the NCBI Taxonomy merges synonyms under the same taxonID, which can be problematic if there is disagreement about whether two taxa are truly synonymous.

**Example**: *Calamagrostisstricta*, formerly NCBI:txid497295, has been merged as a synonym of *Calamagrostisneglecta* NCBI:txid395286 in the NCBI Taxonomy. Furthermore, the GBIF Backbone accepted *C.stricta* (2704899) while designating *C.neglecta* (4104731) as a synonym of *Achnatherumcalamagrostis* (4142326).

**Action**: Searching the NCBI website for a merged taxonID or entering its URL will auto-redirect to the current accepted one. However, the ENA Taxonomy API (https://www.ebi.ac.uk/ena/taxonomy/rest/), which, in principle, uses the same NCBI Taxonomy, usually returns no result for merged taxonIDs, indicating that merged taxonIDs may cause problems with downstream tools that do not take them into account. The currently accepted NCBI taxonID can be added to the Wikidata entry, but the old taxonID may help disambiguate the record and should not be deleted, but, instead, explicitly marked as a deprecated value (see point 1 above).

*3. Incorrect species linked on Wikidata*.

The Wikidata record may be linked to an identifier for a different species. These cases are usually homonyms, which can be recognised by the different taxon author.

**Example**: The Wikidata record for *Rubusgracilis* C.Presl & J.S.Presl (Q17248013) was linked to identifiers for the homonymous *Rubusgracilis* Roxb. in GBIF (2990660) as well as another database, GRIN-Global (32332, explicitly annotated as “non J.S.Presl & C.Presl 1822”).

**Action**: When unambiguous, edit the Wikidata entry to remove the incorrect statement, or point to the correct identifier, if available. Record the access date using the Wikidata property “retrieved” (P813). Different Wikidata items for homonymous taxa can be disambiguated with the property “different from” (P1889).

*4. Ambiguous entry in Wikidata: Conflicting taxon authors*.

Some cases may require taxonomic/nomenclatural expertise or additional information to resolve.

**Example**: The Wikidata record for *Willemetiastipitata* (Q1362051) stated that the taxon author (property P405) is Karl Wilhelm von Dalla Torre (Q79155), but the linked GBIF entry (5389300) for *W.stipitata* (Jacq.) Dalla Torre was annotated as “doubtful” in GBIF, whereas the linked NCBI entry (NCBI:txid519273) represented the homonym *W.stipitata* Cass. Linked records in other Wikis were also inconsistent: German-language Wikipedia – *W.stipitata* (Jacq.) Dalla Torre (https://de.wikipedia.org/wiki/Kronenlattich); Wikispecies – *W.stipitata* Cass. (https://species.wikimedia.org/wiki/Willemetia_stipitata).

**Action**: The Wikidata entity may need to be split into separate entities for each homonym. Start a thread on the corresponding discussion/talk page in Wikidata or Wikispecies to alert other users to the issue. For one’s own research, make a judgement call and document it. Both the GBIF and NCBI records have subsequently been changed, but still disagree on which name should be accepted.

*5. Ambiguous entry in Wikidata: No taxon authors*.

Some taxon names on Wikidata may lack the “taxon author” (P405) or “taxon author citation” (P6507) properties.

**Action**: As above. These should probably be split into separate entities if they are indeed homonyms, but it would then be unclear how the linked identifiers should be distributed between them.

*6. Error in accepted taxon in GBIF Backbone Taxonomy*.

These can often be traced back to errors in the source datasets used to populate the GBIF Backbone. The following example was found because the Wikidata entry was linked to both GBIF and NCBI taxonIDs and agreed with the name-matching with Gndiff, but the author names conflicted.

**Example**: “*Primulamatthioli* K.Richt.” was an accepted taxon in the GBIF Backbone Taxonomy (5640570); GBIF’s source dataset or this name is “Synonymic checklists of the vascular plants of the world” (Hassler 2022). However, the International Plant Names Index (IPNI), a nomenclatural database for botanical names, only reported “*Primulamatthioli* (L.) V.A.Richt.” (https://www.ipni.org/n/702251-1). Wikidata recorded the same author as IPNI for *Primulamatthioli* (Q50859720), namely Vincenz Aladár Richter (Q6163148). GBIF annotated “*Primulamatthioli* (L.) V.A.Richt.” (9764749) as a “homotypic synonym” and additionally had a record for “*Primulamatthioli* (L.) J.A.Richt. 1894” (9781637), also listed as a “homotypic synonym”.

Given the corroboration from IPNI, the author names in GBIF records 5640570 (“K.Richt.”) and 9781637 (“(L.) J.A.Richt.”) are likely to be typographical errors for 9764749 (“(L.) V.A.Richt.”).

**Action**: Report errors or issues via the feedback system on the GBIF website (must be logged in with a GBIF user account). Feedback reports are handled via the issue tracker on GitHub and can also be submitted directly there or by email. The issue opened for the above example is here: https://github.com/gbif/portal-feedback/issues/4673. If their data curators can trace the issue to an upstream data source, the report is passed upwards. Curators can also apply “patches” to the GBIF Backbone Taxonomy, where the upstream source cannot be updated in a timely manner. The GBIF record with the correct authorship (9764749) now has status "accepted".

*7. Error in accepted taxon in NCBI Taxonomy*.

**Example**: *Carexbinervis* Sm. (Wikidata Q160245) was an accepted taxon in the GBIF Taxonomy (2723521), but the NCBI record had different authors “Gren. & Godr.” (NCBI:txid372257) (this has now been corrected).

IPNI listed four homonyms for the name *Carexbinervis*, but none with “Gren. & Godr.” as authors. Only *C.binervis* Sm. was validly published (https://www.ipni.org/?q=carex%20binervis). The remainder were either nom. inval., *C.binervis* Wahlenb. ex Kunth or nom. illeg., *C.binervis* Willd. ex Kunth and *C.binervis* Dewey, the latter according to Plants of the World Online (https://powo.science.kew.org/taxon/urn:lsid:ipni.org:names:77237975-1).

“*Carexbinervis* Gren. & Godr.” turned out to be a chresonym, where the authors after the binomen are not the authors of the name itself, but a reference to a usage of the name in some other publication. The Tropicos database had an entry for “*C.binervis* Gren. & Godr.” with a citation to the publication *Flore de France* by Grenier & Godron (1855) (http://legacy.tropicos.org/Name/9900008). This allowed us to find a digital copy online (https://bibdigital.rjb.csic.es/idviewer/10272/430) where the name “*C.binervis* Sm.” was listed, showing that this was, indeed, the intended name.

Where did the NCBI Taxonomy find this chresonym “*Carexbinervis* Gren. & Godr.”? The NCBI web interface listed two references: Monocot Checklist (http://www.kew.org/wcsp/home.do, accessed 01 Nov 2010) and a research paper (Villaverde et al. 2020). However, the former website is defunct and redirected to Plants of the World Online, which cited only *Carexbinervis* Sm. (Supplementary Table S10). The incorrect taxon authors were presumably sourced from Tropicos or another database which has since been updated or taken offline. Chresonyms look like legitimate taxon names with authorships and cannot be easily detected without cross-checking or conferring original sources, so are especially prone to being propagated across aggregators.

**Action**: Report errors and updates to the NCBI helpdesk by email (Schoch et al. 2020). The example above was corrected shortly after such a report.

*8. Disagreements in taxon concepts between databases*.

The “same” taxon may appear under different names, classifications or even be split or lumped into different taxa, depending on the source consulted. One name may hence represent different taxonomic concepts. When data aggregators designate accepted names or use a particular classification, they gloss over potentially valid taxonomic conflicts (Franz and Sterner 2018).

**Example**: The species *Rosainodora* Fr. (GBIF taxonID 3002258, Wikidata Q15844731) in our dataset did not have an NCBI taxonID, i.e. no sequence data were available. However, *Rosaelliptica* Tausch (GBIF taxonID 3003248, Wikidata Q9325795), listed as a synonym of *Rosainodora* by GBIF, did have an NCBI taxonID (NCBI:txid323240).

**Action**: For our own analyses, we may accept a particular taxonomic opinion and link these taxa that were designated as synonyms by GBIF or NCBI. However, in Wikidata, the NCBI taxonID of *Rosaelliptica* should not be linked from the *Rosainodora* item, but from *Rosaelliptica*. Designation of a synonym is a taxonomic theory which is subject to potential disagreement and future revision. Therefore, the original name of interest, accepted names and synonyms are kept in separate data columns in our workflow. In Wikidata, synonymous taxa can be represented by the “taxon synonym” property (P1420), whereas homonyms can be disambiguated with the “different from” property (P1889).

## Data resources

The above workflow is available from https://github.com/monagrland/taxo-harmo (archived version: https://doi.org/10.5281/zenodo.10074668). Software dependencies are specified in a definition file for the Conda environment manager, using packages distributed via the open-source conda-forge and bioconda channels (Grüning et al. 2018). Code to reformat the input, perform initial name-matching with Gndiff, query Wikidata for identifiers and prepare tables for manual curation is listed and documented in a Jupyter notebook. The workflow can be applied to other GBIF species list datasets simply by updating the filenames and the target taxon group (if not Viridiplantae) and can likewise be re-run with newer versions of the source databases.

## Conclusions

The state of biodiversity identifier linking is patchy, even across well-resourced, heavily used databases and for well-studied sets of species like the German vascular plant flora. As expected, naive name-matching alone is problematic and can cause linking errors, affecting at least 2.8% of Wikidata entries for the species names in the dataset examined here. Ironically, better studied groups and more comprehensive databases may contain more historical names and homonyms that need to be accounted for. Most of such linking errors are easily caught by using author names and higher taxa to disambiguate taxa, allowing us to focus manual curation efforts on the most challenging cases.

Existing recommendations and workflows for taxon name harmonisation (Jin and Yang 2020, Grenié et al. 2021) recognise the same pitfalls of name-matching and the limitations of source databases, such as different accepted synonyms, inconsistent classifications and lack of taxon author citations in some datasets. Dealing with the name-matching problem is by no means straightforward, as evidenced by the infrastructure and numerous tools built by the Global Names Architecture (Gnames) project (Pyle 2016, Mozzherin et al. 2017, Thessen et al. 2022), including the Gndiff tool used in this workflow.

Generally, though, databases are presented as resources to be accepted as-is, over which the user has no influence. Apart from simply filtering out problematic records, what more can be done? We, therefore, suggest the following additional recommendations for users to be active participants and help “pay it forward” in the community:

- Pay attention to potential synonyms and other taxonomic or nomenclatural issues when designing a workflow and choose software tools that can handle them, for example, taxadb (Norman et al. 2020) or tools from Gnames.
- When publishing your own checklists, do not omit taxon authors and higher classification, even when these details appear to be obvious from context.
- Report errors in source databases, as described in the examples above. Both GBIF and NCBI have workflows for dealing with such reports and have been responsive to constructive feedback, in our experience.
- Publish validated, linked identifiers on Wikidata. Each user will, of course, need to check for themselves, but it helps subsequent users filter cases during data-linking to focus manual curation on the more problematic records. The Wikidata data model is highly extensible, so it is possible to perform sophisticated queries and integrate information about taxa with other domains.

Name-matching and identifier-linking are receiving renewed attention from database maintainers. A recent symposium at the TDWG2023 conference touched upon issues raised in this case study from their perspective, such as the importance of identifier mapping to data integration (Fuchs et al. 2023). To complement and harness active user participation, we also suggest that maintainers and developers:

- Establish a transparent channel for user feedback, preferably using an issue tracking system like GitHub (used by GBIF);
- Display version numbers and change logs for individual records. Version control is likely already used internally, but versioning should be exposed to users so that their work is also reproducible. A user should be able to cite (and retrieve) specific record versions via the web site or the API and view the history of changes to a record. In this workflow, we downloaded date-stamped data dumps, but this is not realistic for a casual user looking up individual records and does not reflect intervening changes. There is evidently demand for such information, for example, a third-party project to track changes to the NCBI Taxonomy by analysing dump files (https://github.com/shenwei356/taxid-changelog, Shen and Ren (2021));
- Publish mappings to other database identifiers when available and make them programmatically queryable. The NCBI Taxonomy, for example, displays “LinkOuts” to external databases and citations to taxonomic literature on its web interface, but these are currently not included in the structured data returned via the Entrez API;
- Implement checks against homonyms, for example, by matching authors or comparing higher taxa and flag potential homonyms for verification. This applies to Wikidata contributors writing scripts for automatic import of taxonomic data (“bots”), but also to aggregators like GBIF, which builds its Backbone Taxonomy from third-party data. Some misassigned homonyms, such as *Ammophila* in this case study, can be traced to datasets where the higher taxa were not explicitly specified in the original source. Homonym checks are especially important for name-matching services, which should note when insufficient information is supplied by the user to disambiguate more than one possible name, as done by the GBIF Species Lookup tool.

As a user, why take the trouble to edit Wikidata and send feedback? Curation of biodiversity data is labour-intensive and requires a highly speciali-ed skill-set, so updating community resources will reduce duplicated effort and have a positive, compounding effect (“virtue propagation”). Wikidata, in particular, is increasingly integrated into the biodiversity informatics infrastructure, de facto recognition of its practical usefulness: the database cross-references displayed on species pages on the GBIF website (https://www.gbif.org/species/search) are sourced from Wikidata and the iNaturalist citizen-science app uses Wikidata to link species pages to their respective Wikipedia articles in various languages (Waagmeester et al. 2019). Applications beyond biodiversity show its versatility. Communities can be built on top of Wikidata to curate specific knowledge domains, such as gene annotations (Putman et al. 2017); alternatively, existing wiki-type projects can be imported and interlinked with Wikidata to foster data integration (Martens et al. 2021).

The workflow presented here still relies on ad hoc scripting, which is, to some extent, unavoidable because the point of manual curation is to handle what automation cannot deal with, but it is desirable to minimise this to improve reproducibility, as well as the reusability of code. A promising alternative is OpenRefine (https://openrefine.org/), a dedicated tool for data reconciliation, which records all data-cleaning steps in a given project, allowing them to be shared and re-run on new data. It also supports querying and editing Wikidata within the software, as well as URL-based queries (e.g. calls to the GBIF name parser API). The Simple Standard for Sharing Ontological Mappings (SSSOM, Matentzoglu et al. (2022)) could be used to document the curation and credit the curators involved. The criteria for taxon name-matching in this workflow were defined ad hoc, but could also be formalised with a standardised vocabulary, such as SEMAPV (http://doi.org/10.5281/zenodo.7672104). For example, matching of author names that differ in whether they have diacritics are instances of the SEMAPV term “diacritics suppression”.

Routine sharing of curation workflows by researchers, coupled with the transparent handling of issue reports by database maintainers, will foster more community buy-in and faster adoption of useful practices, improving the quality of downstream analyses.

## Figures and Tables

**Table 1.** Possible outcomes of data-linking steps, further curation steps to be taken and the number of cases identified in this example dataset.

**Name match type**	**GBIF ID linked to Wikidata item**?	**Wikidata links GBIF to NCBI ID**?	**NCBI ID from name matching same as on Wikidata**?	**Wikidata links NCBI to GBIF ID**?	**Taxonomic status of name on GBIF**	**Curation action to take**	**Count**
none	no	-	-	no	-	(a) No matches, including synonyms	1310
none	no	-	-	yes		Other	8
exact	yes	yes	yes	-	-	(b) Match ok, accept automatically	3130
exact	yes	yes	no	-	-	(c) Verify and update NCBI taxonID in Wikidata item	11
exact	yes	no	-	no	-	(d) Batch-add NCBI taxonID to Wikidata item	177
exact	yes	no	-	yes	-	Other	52
exact	no	-	-	yes	“accepted”	(e) Batch-update GBIF taxonID in Wikidata item	245
exact	no	-	-	yes	not “accepted”	(f) Verify if synonym listed in GBIF is valid before linking identifiers	89
noauthor	yes	yes	yes	-	-	(g) Verify if authorships match before linking identifiers	211
noauthor	yes	yes/no	no	-	-	(h) Possible homonym, investigate further	224
author mismatch	yes	yes	yes	-	-	(g) Verify if authorships match before linking identifiers	271
author mismatch	yes	yes/no	no	-	-	(h) Possible homonym, investigate further	217
fuzzy	-	-	-	-	-	other	100

## References

[B10542764] Naturschutz Bundesamt für (2021). Flora von Deutschland (Phanerogamen).

[B10542756] Christen Peter (2012). Data Matching: Concepts and techniques for record linkage, entity resolution, and duplicate detection.

[B10542772] Delpeuch Antonin, Pohl Adrian, Steeg Fabian, Guidry Thad (2023). “Reconciliation Service API v0.1: A protocol for data matching on the web.” W3C Community Group Final Report.

[B10542780] Feng Xiao, Enquist Brian J., Park Daniel S., Boyle Brad, Breshears David D., Gallagher Rachael V., Lien Aaron (2022). A Review of the heterogeneous landscape of biodiversity databases: Opportunities and challenges for a synthesized biodiversity knowledge base. Global Ecology and Biogeography.

[B10542802] Franz Nico M., Sterner Beckett W. (2018). To increase trust, change the social design behind aggregated biodiversity data. Database.

[B10788553] Fuchs Anne, Whitbread Greg, Cooper Endymion (2023). Australian National Species List: Name Identifier Management and Linkages. Biodiversity Information Science and Standards.

[B10542811] Secretariat GBIF (2022). GBIF Backbone Taxonomy. Checklist dataset..

[B10542819] Grenié Matthias, Berti Emilio, Carvajal-Quintero Juan, Dädlow Gala Mona Louise, Sagouis Alban, Winter Marten (2021). Harmonizing taxon names in biodiversity data: A review of tools, databases and best practices. Methods in Ecology and Evolution.

[B10542830] Groom Quentin, Bräuchler Christian, Cubey Robert, Dillen Mathias, Huybrechts Pieter, Kearney Nicole, Klazenga Niels (2022). The disambiguation of people names in biological collections.. Biodiversity Data Journal.

[B10542854] Grüning Björn, Dale Ryan, Sjödin Andreas, Chapman Brad A., Rowe Jillian, Tomkins-Tinch Christopher H., Valieris Renan, Köster Johannes (2018). Bioconda: Sustainable and comprehensive software distribution for the life sciences. Nature Methods.

[B10542869] Hassler Michael (2022). Synonymic Checklists of the Vascular Plants of the World.

[B10542877] Nomenclature International Commission on Zoological (1999). International Code of Zoological Nomenclature.

[B10542885] Jin Jing, Yang Jun (2020). BDcleaner: A workflow for cleaning taxonomic and geographic errors in occurrence data archived in biodiversity databases. Global Ecology and Conservation.

[B10542905] Martens Marvin, Ammar Ammar, Riutta Anders, Waagmeester Andra, Slenter Denise N., Hanspers Kristina, Miller Ryan A. (2021). WikiPathways: Connecting communities. Nucleic Acids Research.

[B10755181] Matentzoglu Nicolas, Balhoff James P, Bello Susan M, Bizon Chris, Brush Matthew, Callahan Tiffany J, Chute Christopher G, Duncan William D, Evelo Chris T, Gabriel Davera, Graybeal John, Gray Alasdair, Gyori Benjamin M, Haendel Melissa, Harmse Henriette, Harris Nomi L, Harrow Ian, Hegde Harshad B, Hoyt Amelia L, Hoyt Charles T, Jiao Dazhi, Jiménez-Ruiz Ernesto, Jupp Simon, Kim Hyeongsik, Koehler Sebastian, Liener Thomas, Long Qinqin, Malone James, McLaughlin James A, McMurry Julie A, Moxon Sierra, Munoz-Torres Monica C, Osumi-Sutherland David, Overton James A, Peters Bjoern, Putman Tim, Queralt-Rosinach Núria, Shefchek Kent, Solbrig Harold, Thessen Anne, Tudorache Tania, Vasilevsky Nicole, Wagner Alex H, Mungall Christopher J (2022). A Simple Standard for Sharing Ontological Mappings (SSSOM). Database.

[B10542918] Mozzherin Dmitry Y., Myltsev Alexander A., Patterson David J. (2017). ‘Gnparser’: A powerful parser for scientific names based on parsing expression grammar. BMC Bioinformatics.

[B10542938] Norman Kari E. A., Chamberlain Scott, Boettiger Carl (2020). Taxadb: A high-performance local taxonomic database interface. Methods in Ecology and Evolution.

[B10542947] Page Roderic D. M. (2022). Wikidata and the Bibliography of Life. PeerJ.

[B10542956] Patterson David, Mozzherin Dmitry, Shorthouse David, Thessen Anne (2016). Challenges with using names to link digital biodiversity iInformation. Biodiversity Data Journal.

[B10542965] Putman Tim E., Lelong Sebastien, Burgstaller-Muehlbacher Sebastian, Waagmeester Andra, Diesh Colin, Dunn Nathan, Munoz-Torres Monica (2017). WikiGenomes: An open web application for community consumption and curation of gene annotation data in Wikidata. Database: The Journal of Biological Databases and Curation.

[B10542978] Pyle Richard (2016). Towards a global names architecture: The future of indexing scientific names. ZooKeys.

[B10542987] Remsen David (2016). The use and limits of scientific names in biological informatics. ZooKeys.

[B10635975] Rutz Adriano, Sorokina Maria, Galgonek Jakub, Mietchen Daniel, Willighagen Egon, Gaudry Arnaud, Graham James G, Stephan Ralf, Page Roderic, Vondrášek Jiří, Steinbeck Christoph, Pauli Guido F, Wolfender Jean-Luc, Bisson Jonathan, Allard Pierre-Marie (2022). The LOTUS initiative for open knowledge management in natural products research. eLife.

[B10543004] Schellenberger Costa David, Boehnisch Gerhard, Freiberg Martin, Govaerts Rafaël, Grenié Matthias, Hassler Michael, Kattge Jens, Muellner‐Riehl Alexandra N., Rojas Andrés Blanca M., Winter Marten, Watson Mark, Zizka Alexander, Wirth Christian (2023). The big four of plant taxonomy – a comparison of global checklists of vascular plant names. New Phytologist.

[B10543022] Schoch Conrad L, Ciufo Stacy, Domrachev Mikhail, Hotton Carol L, Kannan Sivakumar, Khovanskaya Rogneda, Leipe Detlef, Mcveigh Richard, O’Neill Kathleen, Robbertse Barbara, Sharma Shobha, Soussov Vladimir, Sullivan John P, Sun Lu, Turner Seán, Karsch-Mizrachi Ilene (2020). NCBI Taxonomy: a comprehensive update on curation, resources and tools. Database.

[B10543043] Shafee Thomas, Mietchen Daniel, Lubiana Tiago, Jemielniak Dariusz, Waagmeester Andra (2023). Ten quick tips for editing Wikidata. PLOS Computational Biology.

[B10756100] Shen Wei, Ren Hong (2021). TaxonKit: A practical and efficient NCBI taxonomy toolkit. Journal of Genetics and Genomics.

[B10543062] Thessen Anne E., Poelen Jorrit H., Collins Matthew, Hammock Jen (2018). 20 GB in 10 minutes: a case for linking major biodiversity databases using an open socio-technical infrastructure and a pragmatic, cross-institutional collaboration. PeerJ Computer Science.

[B10543053] Thessen Anne, Mozzherin Dmitry, Shorthouse David, Patterson David (2022). Improving the discoverability of biodiversity data using the Global Names Finder. Biodiversity Information Science and Standards.

[B10543071] Turland Nicholas J., Wiersema John H., Barrie Fred R., Greuter Werner, Hawksworth D. L., Herendeen Patrick S., Knapp Sandra (2018). International Code of Nomenclature for Algae, Fungi, and Plants (Shenzhen Code): Adopted by the Nineteenth International Botanical Congress Shenzhen, China, July 2017.

[B10543082] Villaverde Tamara, Jiménez-Mejías Pedro, Luceño Modesto, Waterway Marcia J, Kim Sangtae, Lee Bora, Rincón-Barrado Mario, Hahn Marlene, Maguilla Enrique, Roalson Eric H, Hipp Andrew L, Wilson K L, Larridon I, Gebauer S, Hoffmann M H, Simpson D A, Naczi R F C, Reznicek A A, Ford B A, Starr J R, Park J, Escudero M, Martín-Bravo S (2020). A new classification of *Carex* (Cyperaceae) subgenera supported by a HybSeq backbone phylogenetic tree. Botanical Journal of the Linnean Society.

[B10543156] Vrandečić Denny, Krötzsch Markus (2014). Wikidata. Communications of the ACM.

[B10543112] Waagmeester Andra, Mietchen Daniel, Leachman Siobhan, Groom Quentin (2019). Using crowd-curation to improve taxon annotations on the Wikimedia infrastructure. Biodiversity Information Science and Standards.

[B10543121] Waagmeester Andra, Stupp Gregory, Burgstaller-Muehlbacher Sebastian, Good Benjamin M, Griffith Malachi, Griffith Obi L, Hanspers Kristina, Hermjakob Henning, Hudson Toby S, Hybiske Kevin, Keating Sarah M, Manske Magnus, Mayers Michael, Mietchen Daniel, Mitraka Elvira, Pico Alexander R, Putman Timothy, Riutta Anders, Queralt-Rosinach Nuria, Schriml Lynn M, Shafee Thomas, Slenter Denise, Stephan Ralf, Thornton Katherine, Tsueng Ginger, Tu Roger, Ul-Hasan Sabah, Willighagen Egon, Wu Chunlei, Su Andrew I (2020). Wikidata as a knowledge graph for the life sciences. eLife.

